# Tunable exciton-polaritons emerging from WS_2_ monolayer excitons in a photonic lattice at room temperature

**DOI:** 10.1038/s41467-021-24925-9

**Published:** 2021-08-16

**Authors:** L. Lackner, M. Dusel, O. A. Egorov, B. Han, H. Knopf, F. Eilenberger, S. Schröder, K. Watanabe, T. Taniguchi, S. Tongay, C. Anton-Solanas, S. Höfling, C. Schneider

**Affiliations:** 1 Technische Physik and Wilhelm-Conrad-Röntgen-Research Center for Complex Material Systems, Universität Würzburg, Würzburg, Germany; 2 Institute of Physics, University of Oldenburg, Oldenburg, Germany; 3 Institute of Condensed Matter Theory and Solid State Optics, Friedrich Schiller University, Jena, Germany; 4 Institute of Applied Physics, Abbe Center of Photonics, Friedrich Schiller University, Jena, Germany; 5 Fraunhofer-Institute for Applied Optics and Precision Engineering IOF, Jena, Germany; 6 Max Planck School of Photonics, Jena, Germany; 7 Research Center for Functional Materials, National Institute for Materials Science, Tsukuba, Japan; 8 International Center for Materials Nanoarchitectonics, National Institute for Materials Science, Tsukuba, Japan; 9 School for Engineering of Matter, Transport, and Energy, Arizona State University, Tempe, AZ USA

**Keywords:** Bose-Einstein condensates, Nonlinear optics

## Abstract

Engineering non-linear hybrid light-matter states in tailored lattices is a central research strategy for the simulation of complex Hamiltonians. Excitons in atomically thin crystals are an ideal active medium for such purposes, since they couple strongly with light and bear the potential to harness giant non-linearities and interactions while presenting a simple sample-processing and room temperature operability. We demonstrate lattice polaritons, based on an open, high-quality optical cavity, with an imprinted photonic lattice strongly coupled to excitons in a WS_2_ monolayer. We experimentally observe the emergence of the canonical band-structure of particles in a one-dimensional lattice at room temperature, and demonstrate frequency reconfigurability over a spectral window exceeding 85 meV, as well as the systematic variation of the nearest-neighbour coupling, reflected by a tunability in the bandwidth of the p-band polaritons by 7 meV. The technology presented in this work is a critical demonstration towards reconfigurable photonic emulators operated with non-linear photonic fluids, offering a simple experimental implementation and working at ambient conditions.

## Abstract (web summary)

Excitons in atomically thin crystals couple strongly with light. Here, the authors observe lattice polaritons in a tunable open optical cavity at room temperature, with an imprinted photonic lattice strongly coupled to excitons in a WS_2_ monolayer.

## Introduction

Exciton-polaritons in lattices have matured to a very promising and versatile platform in applied information technology^1–4^. Their bosonic character enables ultra-fast condensation processes^5–8^, which are already exploited in classical emulation applications^1,2^, and can be also harnessed in ultra-fast quantum annealing architectures^9^. Their intrinsically strong non-linearity has very recently opened the area of genuine quantum polaritonics^10,11^. The combination of exciton-polaritons with photonic lattices, hence, is a powerful approach to the simulation of complex Hamiltonians, with the perspective to explore topologically non-trivial phenomena and possibly quantum effects.

With the emergence of atomically thin crystals and the observation of giant light-matter coupling of excitons hosted in transition metal dichalcogenide (TMDC) monolayers, the research field on exciton-polaritons has experienced a paradigmatic shift on the material side: excitons in TMDC monolayers present a very large oscillator strength^12^ and their large binding energies routinely facilitate the observation of exciton-polaritons from cryogenic up to room temperature^13–16^. At the same time, with the current developments on the material optimization, also their structural homogeneity becomes competitive with high quality epitaxially grown III-V semiconductors^17^. Last but not least, there is a sizeable polariton interaction^18,19^ which can be engineered via injecting electrons or combining various monolayers in a van-der-Waals heterostructure^20^. Those advantages, very recently, led to the first observations of polariton condensation in hybrid TMDC-III-V and in pure TMDC microcavity architectures^21,22^.

In this work, we significantly expand the versatility of TMDC exciton-polaritons, by demonstrating their formation in a one-dimensional photonic lattice imprinted in a so-called open cavity. The structure clearly forms the canonical gapped spectrum of Bloch-polaritons^23–25^, and its “on-the-fly” cavity reconfigurability allows us to demonstrate the emergence of frequency tunable Bloch polaritons, with the advantage of operating at room temperature.

## Results

### Sample and experimental setup

**Fig. 1:**
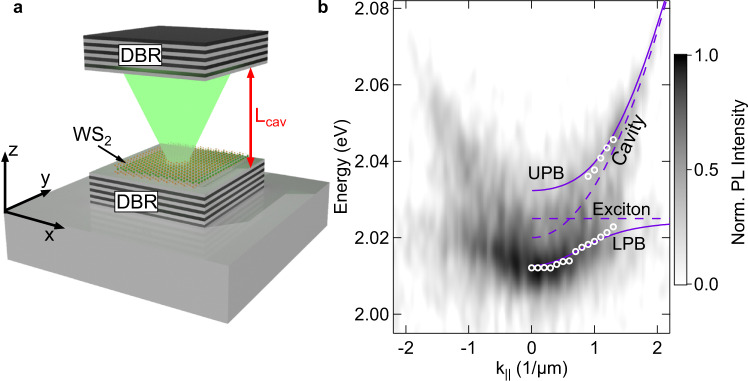
Open cavity and TMDC monolayer sketch and strong coupling conditions. **a** Sketch of the open cavity device, composed of two DBRs embedding an air gap. The WS_2_ monolayer is placed on top of the bottom DBR. **b** Momentum-resolved photoluminescence spectrum of the polariton emission for a *L*_cav_~2.2 µm, under weak excitation with a green, non-resonant, continuous wave laser. As a result of the peak fitting of the UPB and LPB modes, see white circles, the excitonic and photonic modes [UPB and LPB] are represented with dashed [full] lines for k_||_ > 0. The spectrum is recorded with a pump power of ~5 µW and 300 s integration time.

The implementation of our experimental platform is schematically depicted in Fig. 1a. Our device is composed of two distributed Bragg reflectors (DBRs) of SiO_2_/TiO_2_ (both top and bottom mirrors containing 10 mirror pairs), which are separated by an air-gap. Both DBRs are attached to an XYZ set of nano-positioners. The two mirrors form an open cavity, whose cavity resonance is tunable on-demand by varying the DBRs separation with nm-precision. The atomically thin WS_2_ layer is isolated from a flux-grown bulk crystal, and transferred onto the bottom DBR via the dry-gel stamping method^26^ (more details about the optical properties of the monolayer and the open cavity setup can be found in the Supplementary Note 1).

Since the bottom DBR is terminated on a low index material of $\lambda /(4n)$ thickness (SiO_2_), the monolayer will permanently remain in the optical field antinode, and thus optimally coupled to the longitudinal optical cavity modes.

It is possible to assess the physical distance between the two DBR mirrors by analyzing the separation of the longitudinal optical cavity modes and comparing those to transfer matrix simulations (see Supplementary Note 2). From those studies, we find that our mirrors can be approached to a distance of ~2 µm.

Next, at a mirror distance of 2.2 ± 0.5 µm, we study the light-matter coupling conditions in our device by inspecting the dispersion relation of the polariton photoluminescence under weak excitation with a continuous wave green laser (see Methods for more details), see Fig. 1b. The longitudinal resonance mode of the open cavity (${E}_{C}=2.020$ eV) is spectrally tuned to resonance with the exciton energy of the WS_2_ monolayer (${E}_{X}=2.025$ eV), with a negative detuning of $\triangle ={E}_{C}-{E}_{X}=-5$ meV. At resonance conditions, the emitted cavity photoluminescence deviates from the standard parabolic dispersion relation of an empty cavity and breaks into two upper and lower polariton branches (UPB and LPB). We analyze the peak position of the respective polariton modes by iterative fitting, see white circles in Fig. 1b. In order to determine the Rabi splitting, we apply a standard coupled oscillator approach (see Supplementary Note 3), reflecting strong coupling conditions in our device with a splitting of $\Omega =19\pm 3$ meV. The exciton vs. photon admixture of the polaritonic modes can be conveniently controlled by changing the separation of the two DBR mirrors, yielding an excellent control of the relevant polaritonic properties in our open cavity, as we demonstrate in the Supplementary Note 4. It is worth noting, that a similar magnitude of the Rabi-splitting has been previously found in TMDC-loaded open cavities of similar effective length^14^, and it is in full agreement with our numerical simulations using the transfer matrix method (see Supplementary Note 2).

### Room temperature TMDC Bloch-polaritons in an open cavity

To engineer the confinement of TMDC-polaritons, we utilize focused Gallium ion beam (FIB) milling to mechanically shape hemispheric traps of 5 µm diameter and an approximate depth of ~350 nm in a glass carrier; the top DBR is deposited in the last step via dielectric sputtering. It is worth noting, that the sputtering process does not yield a planarization, or a significant roughening of the FIB structure.

**Fig. 2:**
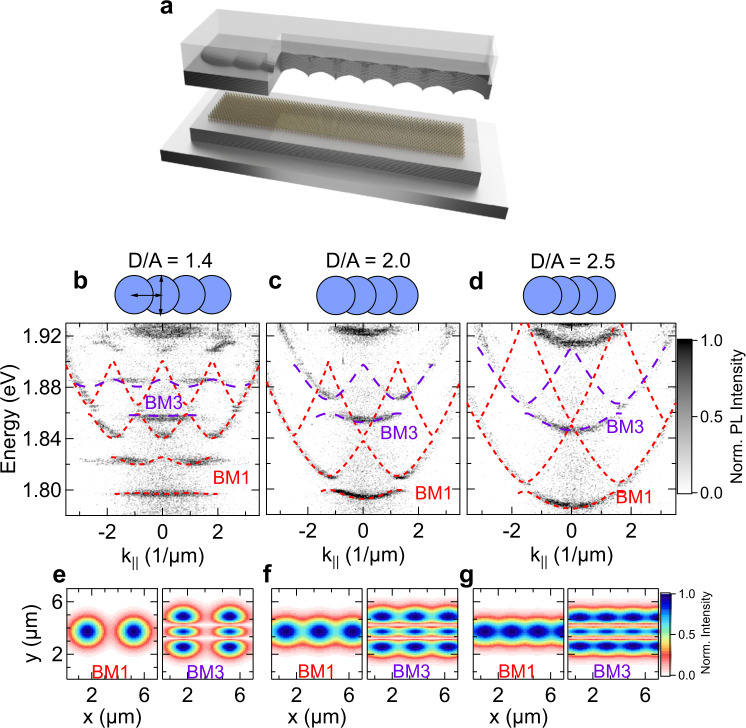
Optical properties of TMDC polaritons in a lattice. **a** Sketch of the open cavity device with the 1D photonic lattice implemented in the top mirror. **b**–**d** Momentum-resolved photoluminescence spectrum from one-dimensional polariton lattices (see the sketch of the photonic potential in the top part of each panel) with increasing diameter (vertical arrow) to center-center distance (horizontal arrow) ratio *D/A* = 1.4, 2.0 and 2.5, respectively. The dispersion relation maps are encoded in a normalized, false-colour scale. The dashed lines are theory fittings to the experimental data. Experimental data are recorded with a pump power of ~100 µW and an integration time of 480 s. **e**–**g** Profiles of the Bloch modes with one (BM1) or three field maxima (BM3) in transverse to the lattice structure.

Polariton trapping in single hemispheric traps manifests in a discretized dispersion, giving rise to well-defined confined modes labelled s, p, and d in this paper (see Supplementary Fig. 8). In the presence of a neighbouring trap with sufficiently strong overlap, we can witness the formation of molecular modes, reflected by a molecular splitting most notable in the s- and the d-state (Supplementary Fig. 8). By utilizing this accurate control of mode-coupling, we create one-dimensional lattice structures with varying inter-trap distance (*A*) and *D* is the trap diameter(*D = *5 µm, for the experiments shown here). In the open cavity configuration, this yields tunable lattices, as sketched in Fig. 2a, as well as schematically depicted in the top parts of Fig. 2b–d.

Figure 2b–d show the polariton dispersion relation, which was recorded via momentum-resolved photoluminescence measurements of three different lattices, with traps of *D = *5 µm diameter and varying overlap *A*, *D/A = *1.4, 2.0, and 2.5, respectively. The full control on the spatial configuration of the open cavity allows studying different photonic potentials with the same monolayer, by laterally displacing the top DBR containing the imprinted lattices at different lateral positions.

The photoluminescence maps clearly reveal the formation of a characteristically gapped massive-particle dispersion relation of polaritons in a lattice. The magnitude of the energy gap, which separates the photonic bands emerging from the coupling of s- and p-type orbitals, as well as the curvature of the bands, critically depends on the nearest-neighbour hopping in the photonic lattice which is determined by the *D/A* ratio.

While the curvature of the polariton bands strongly increases for increasing nearest-neighbour overlap (*D/A*), the size of the photonic gap, which emerges at the edge of the one-dimensional Brillouin zone, decreases from 26 ± 2 to 4 ± 2 meV. In order to provide a deeper and more quantitative understanding of our system, we model the dispersion relation as well as the Bloch modes emerging in our system in the framework of a mean-field model with effective potential (dashed lines in Fig. 2b–d). Details on the model are found in the Supplementary Note 6. Our model reveals nearest-neighbour hopping constants of ~0.2–2.7 meV, which are of similar magnitude as in previous works utilizing coupled hemispheric cavities with organic materials^27^. The corresponding real-space profile of Bloch-modes yielding the s-band formation (BM1) is plotted in Fig. 2e–g.

It is worth noting, that the emergence of a second Bloch band, approx. 50 meV blue-shifted from the ground-mode (at 1.85 meV in Fig. 2b, the theory is plotted in dashed purple lines) is not related to a higher longitudinal mode. It can be associated with the band structure arising from photonic orbitals of d-symmetry, coupling in the π-configuration. The real-space profile of the Bloch-modes (BM3), featuring three maxima in the transverse direction to the one-dimensional lattice, is plotted in Fig. 2e–g.

In contrast, only the sigma-coupling of the p-orbitals can be seen in our spectra (the second band, dashed red lines).The π-coupling of p-orbitals (Supplementary Fig. 9, BM2), featuring an even number of field maxima in transverse direction to the lattice, as expected, remain dark for a pump-spot oriented in the center of the lattice and should only significantly contribute to the signal for a strongly misplaced excitation pump. The excellent agreement between experiment and theory finally allows us to directly assess a physical cavity length of ~2.2 µm.

### Tunablity of the polariton lattice

While Bloch band polaritons in microcavity lattices are widely explored in micro-structured GaAs samples at cryogenic conditions^13^, and more recently in organic and perovskite structures at ambient conditions^27,28^, the remarkable asset of our approach comes along with the intrinsic space- and spectral-tunability provided by the open cavity advantage. The vertical displacement of the micro-structured top DBR changes the optical resonance conditions on demand and consequently controls the eigen-energies of the Bloch–polaritons.

**Fig. 3:**
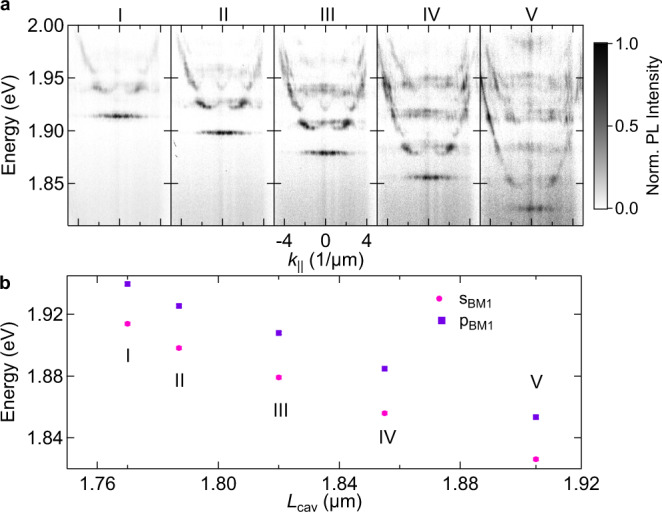
Widely tunable lattice polaritons. **a** Momentum-resolved PL spectra of the 1D linear chain with a lens diameter of *D* = 5 µm and an overlap *D/A* = 1.7 for increasing DBR separations from left to right. The PL emission arises as a result of the coupling between the photonic lattice and the WS_2_ excitons. The pump power is ~ 227 µW and an integration time of 480 is used for each individual spectrum. The PL map intensities are coded in a false colour scale included on the right side of the panels. **b** Dependence of the s- (magenta circles) and p-band (violet squares) energy versus cavity length *L*_cav_. The energy was tuned over 87.8 meV (s-band) 86.3 meV (p-band) with a change of the DBR separation by 135 nm. The error bars in (**b**) represent the accuracy in the energy determination.

Figure 3a depicts the dispersion relation of the one-dimensional linear chain with a diameter of *D = *5 µm and an overlap *D/A = *1.7 for increasing cavity length from left to right, which corresponds to a variation of the cavity length of ~135 nm. The sprectrum in panel I(V) corresponds to a cavity length of 1.770(1.905) µm. The open cavity tunability allows the giant, continuous spectral modification of the polariton band-structure by more than 85 meV, see Fig. 3b, where we depict the energy change of the s- (magenta circles) and p-band (violet squares) versus the cavity length *L*_cav_. The detuning of the s-band energy is varied from −93(I) to −181(V) meV due to the tuned cavity length. The excitonic fraction, represented by the Hopfield coeffiecient *|X* | ^2^, varies from 3.1(I) to 0.6(V) %, accordingly (full information on the detuning and the Hopfield coefficients is given in the Supplementary Note 4, Supplementary Table 2).

**Fig. 4:**
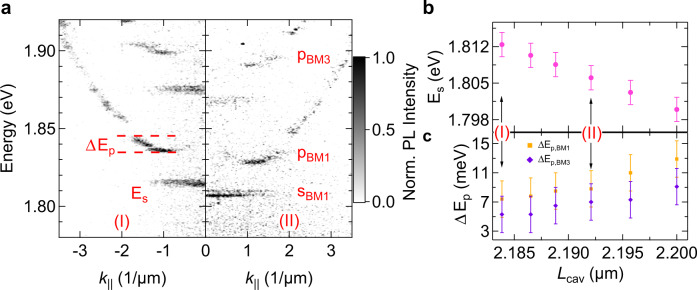
Tunable coupling in a photonic lattice. **a** Momentum-resolved photoluminescence spectrum of a polariton chain with *D/A* = *1.7* for two different DBR separations of 2.184 and 2.192 µm in the regions of k_||_ < 0 (I, left) and k_||_ > 0 (II, right), respectively. The map intensity is coded in a false colour scale included in the right side of the map. Each spectrum is recorded with a pump power of ~ 100 µW and 480 s integration time. **b**, **c** Dependence of the s-band energy (magenta circles) and the p-band bandwidth (for Bloch modes 1 (BM1) and 3 (BM3), presenting an energy difference of 7 meV) versus cavity length. The labels (I, II) mark the corresponding *E*_*s*_ and Δ*E*_*p*_ values of panels in (**a**). The error bars in (**c**, **d**) represent the accuracy in the energy determination.

The momentum-resolved PL spectra for a smaller variation of the cavity length are shown in Fig. 4a: the negative and positive k_||_ regions in panel (a) correspond to a DBR separation of 2.184 and 2.192 µm, respectively. The tuning of the open cavity results in a spectral shift of more than 12 meV, see Fig. 4b.

In addition, the modification of the cavity length yields significant changes in the coupling configuration: We capture a profound modification of the width of the Bloch-bands, which we study for the sigma-coupled p-band (Bloch mode 1, BM1), as well as the π-coupled d-band (Bloch mode 3, BM3). In Fig. 4(c), we plot the extracted bandwidth of these bands as a function of the cavity length. The bandwidth change reflects a tunability of the nearest-neighbour coupling up to 5 meV by nanometrically displacing the top DBR by 16 nm. Such a fine adjustment of nearest-neighbour coupling in a polariton lattice is an inspiring result towards experiments calling for well-balanced on site- and nearest-neighbour coupling configurations.

The emergence of the tunability of photonic hopping can be naively expected to route in a modification of the mode index, as the cavity length is modified. This yields a much slower trend as captured in our experiment (see Supplementary Note 7). However, in our numerical calculations, we found strong indications for the emergence of resonance yielding a very strong dependence between cavity length and nearest-neighbour hopping on the order of our experimental observations.

## Discussion

We have presented an open cavity system with an integrated photonic lattice, strongly coupled to an atomically thin layer of WS_2_ at room temperature. The imprinted lattice geometries in the open cavity leads to a great control and reconfigurability in the engineering of Bloch-polaritons. We demonstrate a giant spectral tunability in energy by more than 85 meV, and a significant tunability of the nearest-neighbour coupling in the lattice. While our experiments are carried out in the linear regime, it is reasonable to assume that the non-linear regime of Bosonic condensation can be reached in a similar approach. The possibility to engineer and increase the non-linearities of TMDC polaritons, e.g. by utilizing van der Waals heterostructures^29^, makes our system highly interesting for applications relying on non-linear photonic lattices.

## Methods

### Sample

In order to minimize the distance between the two mirrors, the GaAs substrate of the bottom mirror is etched forming a mesa with dimensions 200 µm × 200 µm, and 20 µm depth (see Supplementary Note 1). The hemispheric photonic traps of the top mirror are sculpted via FIB in a glass carrier. These traps present a diameter of 5 µm and an approximate depth of ~350 nm. Details on the studied photonic lattices can be found in the Supplementary Note 1. Each DBR is formed via dielectric sputtering. It is worth noting that the sputtering process in the DBR formation does not yield a planarization, or a significant roughening of the FIB structure in the top mirror.

The WS_2_ monolayer (obtained from a CVD-grown bulk crystal) is transferred onto the bottom DBR via the dry-gel stamping method. The optical properties of the WS_2_ monolayer are studied in the Supplementary Note 1. The thickness of each SiO_2_ [TiO_2_] $\lambda /4$-layer is 102.9 [64.6] nm, optimized for a 630 nm cavity resonance. The cavity is terminated in SiO_2_ (lower refractive index) to ensure the maximum coupling of the WS_2_ monolayer to the photonic cavity field.

### Experimental setup

The laser source is a continuous wave, green laser; its pump power is controlled with adjustable optical density filters in the excitation path. The laser is tightly focused in the top mirror of the cavity with an Mitutoyo objective (×50, 0.42 NA). This allows to form a Gaussian spot size with a FWHM diameter of ~3.6 µm. The spectrometer [CCD] used for these experiments is an Andor Shamrock 500i [Andor Newton EMCCD DU971P-BV]. Further details on the experimental setup for the reconstruction of the real- and momentum-space distribution are given in the Supplementary Note 1.

## Supplementary information

Supplementary Information

## Data Availability

The data that support the findings of this study are availiable from the corresponding author upon reasonable request.
