# Supplementary material for: Tunable exciton-polaritons emerging from WS2 monolayer excitons in a photonic lattice at room temperature
Source: Nat Commun. 2021 Aug 16;12:4933. doi: 10.1038/s41467-021-24925-9 (PMC8368091; doi:10.1038/s41467-021-24925-9)
Supplement: Supplementary file 1 — Supplementary Information [file 41467_2021_24925_MOESM1_ESM.pdf]

Supplementary Information

Tunable exciton-polaritons emerging from WS_2 monolayer excitons in a photonic lattice at room temperature

L. Lackner[1,2,†], M. Dusel[1], O.A. Egorov[3], B. Han[2], H. Knopf[4,5,6], F. Eilenberger[4,5,6], S.Schröder[5], K. Watanabe[7], T. Taniguchi[8], S. Tongay[9], C. Anton-Solanas[2], S. Höfling[1] and C. Schneider[1,2,†]

[1]Technische Physik and Wilhelm-Conrad-Röntgen-Research Center for Complex Material Systems, Universität Würzburg, D-97074 Würzburg, Am Hubland, Germany

[2]Institute of Physics, Carl von Ossietzky University, 26129 Oldenburg, Germany.

[3]Institute of Condensed Matter Theory and Solid State Optics, Friedrich Schiller Universität Jena, Max-Wien Platz 1, 07743 Jena, Germany.

[4]Institute of Applied Physics, Abbe Center of Photonics, Friedrich Schiller University, 07745 Jena, Germany.

[5]Fraunhofer-Institute for Applied Optics and Precision Engineering IOF, 07745 Jena, Germany.

[6]Max Planck School of Photonics, 07745 Jena, Germany.

[7]Research Center for Functional Materials, National Institute for Materials Science, 1-1 Namiki, Tsukuba 305-0044, Japan

[8]International Center for Materials Nanoarchitectonics, National Institute for Materials Science, 1-1 Namiki, Tsukuba 305-0044, Japan

[9]School for Engineering of Matter, Transport, and Energy, Arizona State University, Tempe, Arizona 85287, USA

[†]Corresponding authors: lukas.lackner@uol.de, christian.schneider@uol.de

Supplementary Note 1. Sample, experimental open cavity setup and PL imaging system

Tungsten disulfide (WS_2) vdW crystals were grown using a two-step flux technique. As received tungsten (5N purity Alfa Aesar) and sulfur (5N purity Sigma Aldrich) were further purified to 6N purity or higher using electrocatalytic reaction and/or sublimation technique. These high purity powders (300 mesh) were mixed till a uniform mixture was formed using a 14-days process using an automated powder tumbler process. The uniform mixture contained a stoichiometric ratio of W:S (1:2 molar) with access sulfur to ensure a close to 1:2 ratio. The mixture was sealed under 1E-7 Torr pressure in a 2 mm thick quartz ampoule that measured 19 cm in length and baked at high temperatures (1000 °C ramp rate 20 °C / hour, natural cooling) till polycrystalline ~200-100 mesh binary WS_2 vdW powders were created. The usual process required a 1 to 3 weeks annealing process. After the first step, these powders were removed from the ampoule under argon backfilled glovebox and further ground using the tumbler process to create uniform size density vdW WS_2 powders.

In the 2nd step, these powders were sealed in the ampoule without any transport agent under 1E-7 Torr or better pressure with 2 mg/1 g extra sulfur concentration. The access sulfur was essential to obtaining defect-free WS_2 crystals without bound exciton peaks at low temperatures. The sealed ampoule was ramped very slowly to 1210 °C at 20 °C/hour and kept at this temperature for 3 days. One end of the ampoule was gradually dropped to 1170 °C (temperature differential of 40 °C) over 2 days. The entire process (2nd segment) took 5 weeks to yield a few mm-sized self-flux grown crystals without any transport agent aide to limit contamination arising from I_2, Br_2, or other transport agents. In the entire process, the purity

of the powders, formation of high mesh number vdW powders as precursors, careful cold end temperature drop, and the duration of the crystal were essential to achieving excellent excitonic grade WS_2 crystals.

The atomically thin WS_2 layer is mechanically exfoliated from the grown bulk crystal and then transferred onto the bottom DBR via the dry-gel stamping method[1]. After the transfer of the WS_2 monolayer (or few layer hBN) the sample is annealed at 100 °C on a hot plate for 1 min.

To facilitate a micrometric approach of the two distributed Bragg reflector (DBR) mirrors (<10 µm), we have etched a rectangular mesa of dimensions 200 µm x 200 µm, and 20 µm depth into the GaAs substrate, before evaporating the bottom DBR[2] (see Supplementary Figure 1(a,b) where the limits of the mesa are visible). Both, bottom and top DBR consist of 10 pairs SiO_2/TiO_2. The thickness of each SiO_2 [TiO_2] $\lambda/4$-layer is 102.9 [64.6] nm, optimized for a 630 nm cavity resonance. The cavity is terminated in SiO_2 (lower refractive index) to ensure the maximum coupling of the WS_2 monolayer to the photonic cavity field.

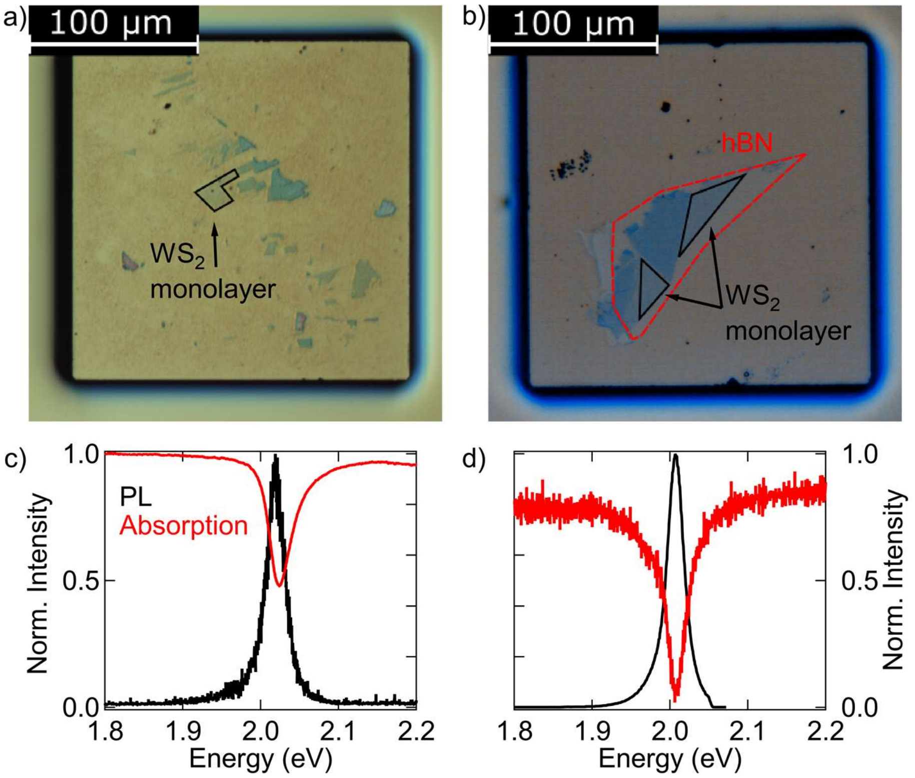

Supplementary Figure 1. PL and absorption of the WS_2 monolayer. (a,b) Microscope images from two different samples: a) WS_2 monolayer (borders delimited with a full black line) is transferred to the bottom DBR. In panel (b) the monolayer is covered with an hBN flake (borders delimited with a dashed red line). (c,d) Corresponding PL emission and WL absorption spectra represented in black and red traces, respectively. In the PL spectra shown in c(d), we use a pump power of ~1(1) µW and 1(30) s integration time. For the WL absorption, an integration time of 30(1) s is used.

The WS_2 monolayer in Supplementary Figure 1a) is of dimensions of 11 µm x 9 µm (small extension 8 µm x 3 µm). For the second device (b), experimental results on the upper monolayer with an extension of ~ 40 µm x 15 µm are presented. Here the monolayer is covered by few layer hBN in order to protect the sample from: (i) mechanical friction between the two DBR mirrors and (ii) degeneration induced by high pump power excitations. In panel c)/d) corresponding PL (black trace) and WL absorption (red trace) spectra are shown. The spectral position of the exciton PL[WL] is 2.019/2.007[2.025/2.007] eV. The corresponding FWHM of these peaks is 25/22[33/28] meV.

In Supplementary Figure 2 microscopy images of the structured top DBR mirror are shown. The hemispheric photonic traps of the top mirror are sculpted via Focused Ion Beam (FIB) in a glass carrier (SCHOTT ‘D 263® T eco Thin Glass’, thickness=550 µm). These traps are of varying diameter and shape. Here only one set of one-dimensional linear chains is shown. Independent on the shape (lens, molecule or chain) the structures have an approximate depth of ~350 nm. And no planarization, or a significant roughening of the FIB structures is induced by the sputtering process.

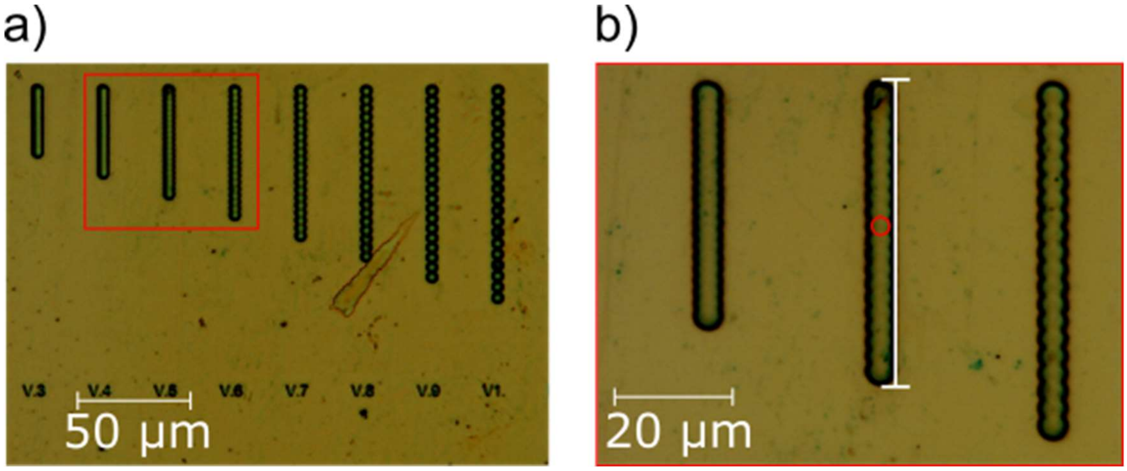

Supplementary Figure 2. Detail on the photonic chains in the top DBR. a) Microscope image of the structured DBR mirror. All the displayed linear chains present a single site diameter of D=5 µm. The overlap V=A/D is varied between 0.3 to 1.0, where A is the center-to-center distance between consecutive lenses. The red rectangle marks a region of interest enlarged in the next panel. b) Magnified view of the marked area in panel a), displaying three linear chains with overlaps V= 0.4, 0.5 and 0.6, respectively. The laser spot size used during the experiments is indicated with a red circle on the chain. The white scales in these panels indicate the spatial dimensions of the images.

In order to realize the air-gap open cavity, both DBR mirrors, are attached to a set of nano-positioners (see Supplementary Figure 3). The bottom DBR is mounted on a stack of five nano-positioners. Going from bottom to top, initially, a XY- (12 mm travel range, model ecsxy5050alnumrt) and a Z-positioner set (8 mm travel range, ecsz5050alnumrt) is placed. Next, the tilt-positioners, (forming a goniometer, see labels θ and φ in panel (b), models ecgt5050alnumrt and ecgp5050alnumrt) and a rotator (rot, providing 360 deg rotation, model ecr3030alnumrt) finalize the full set of motors for the bottom part of the cavity. The height of the monolayer sample is conveniently designed to be placed in center of rotation of the tilt motors. The top DBR is mounted on a replica of the XY- and Z-positioner set previously discussed.

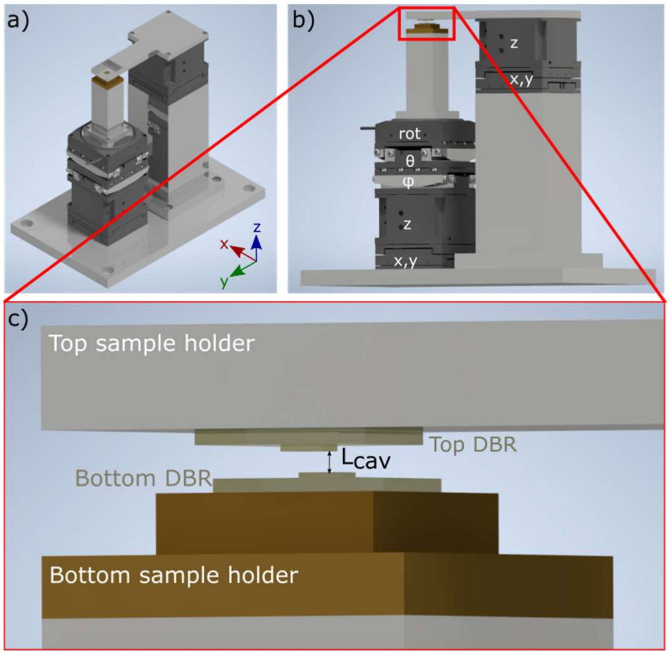

Supplementary Figure 3. CAD-drawing of the air-gap open cavity system. (a,b) Overview of the air-gap open cavity assemble in different perspectives. The red box in panel (b) indicates the area where the sample is loaded in the microcavity. The different labels in panel (b) indicate all the degrees of freedom controlled by the motors in order to align the open cavity system. (c) Enlarged area from previous panel (b). The two DBR mirrors are pasted to individual sample holders (the monolayer is deposited on top of the bottom DBR). The bottom/top sample holder is made out of bronze/steel.

Supplementary Figure 4 shows a sketch of the lens configuration to collect far- (momentum-space) and near-field (real-space) distributions throughout the experiments. For the momentum-resolved measurements a Fourier imaging configuration is used. Lens L_K collects the angle-dependent information of the back-focal plane of the microscope objective L_{Obj} (50x, 0.42 NA). To collect real-space resolved PL an extra lens L_R is inserted in the beam path. Both, near- and far-field, images are projected in the spectrometer focal plane (slit) by L_S.

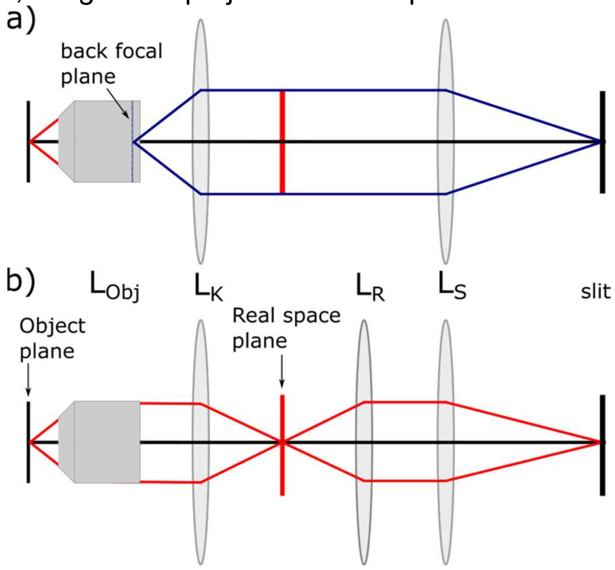

Supplementary Figure 4. Optical system scheme for real- and momentum-space imaging. a) A Fourier imaging configuration is used to create an image in the focus plane of the spectrometer system (slit). The Fourier lens (L_K, f=300 mm) collects the angle-dependent information in the back focal plane of the microscope objective (L_{obj}, ×50, 0.42 NA) which is projected to the spectrometer focal plane by the collection lens (L_S, f= 400mm). b) To collect the real-space distribution PL an extra lens (L_R, f=200 mm) is inserted in the beam path.

Supplementary Note 2. Longitudinal optical cavity modes versus DBR separation and transfer matrix simulations

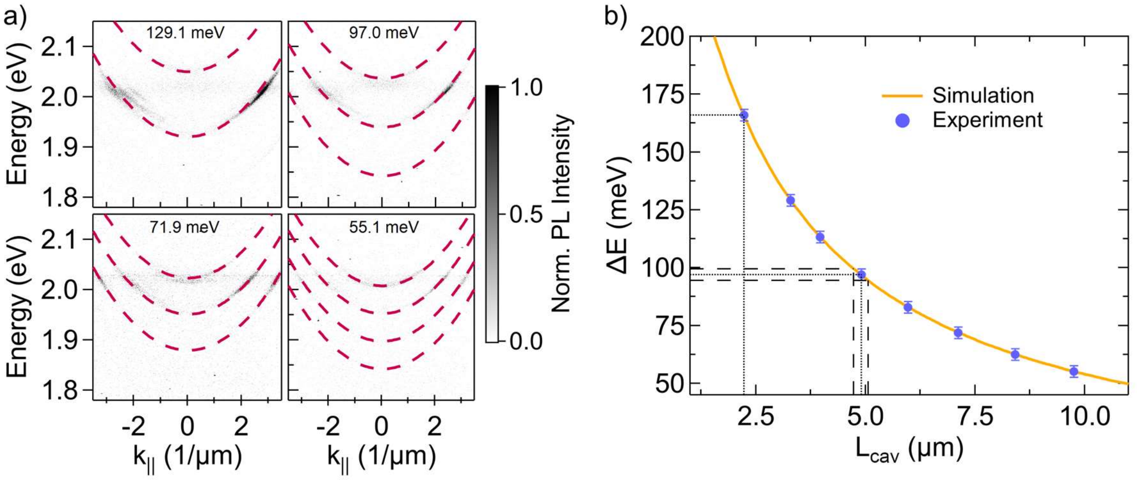

Supplementary Figure 5. Cavity mode energy splitting versus cavity length. (a) Momentum-resolved photoluminescence spectrum of the cavity under different DBR separations, 3.3, 4.9, 7.1 and 9.8 µm, respectively. The different parabolic cavity modes are highlighted with dashed lines. The excitation setting is the same as that indicated for Fig. 1(b) of the main text. (b) Transfer Matrix Simulation of the energy splitting between the cavity modes as a function of DBR separation, the violet circles located on the full line depict the different experimental energy splittings (and therefore the different DBR separations) which have been studied. Pump power ~5 µW and 300 s integration time.

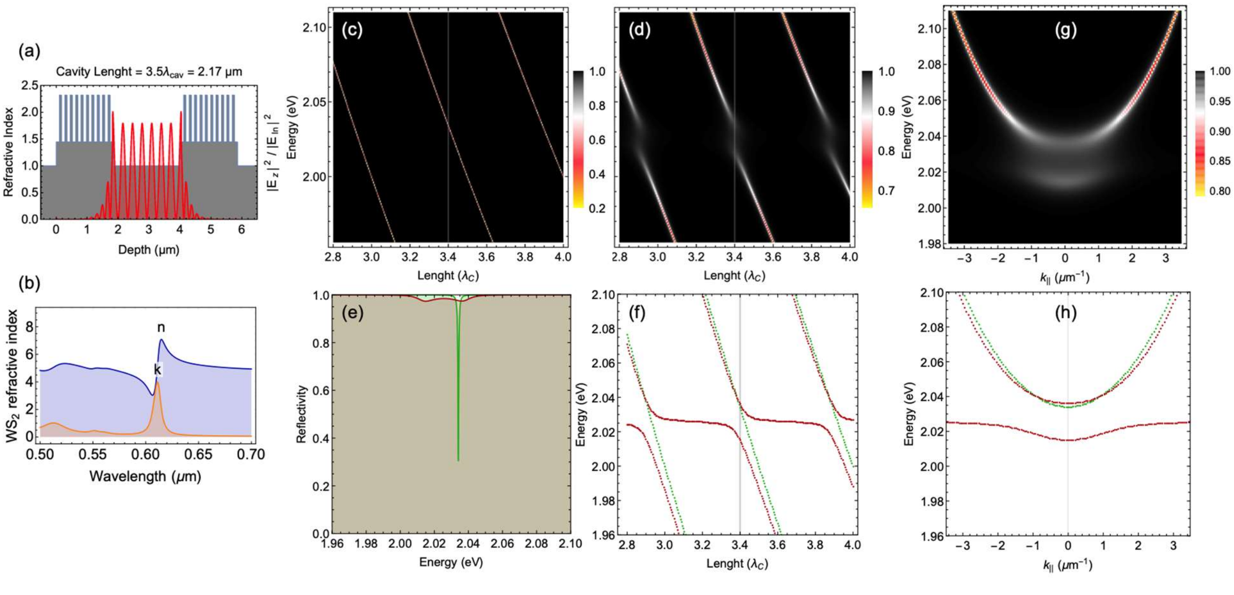

Supplementary Figure 6. Transfer Matrix Method for the simulation of the dispersion relation. (a) Refractive index profile of the cavity (left axis, gray color) and normalised intensity profile of the cavity mode at the cavity resonance $\lambda_C = 0.62\ \mu m$ (right axis, red color) versus cavity depth. The air spacer has been simulated for a cavity length of $L_C = \lambda_C$. The cavity configuration presents a maximum in the place where the WS_2 monolayer is deposited (bottom mirror surface). (b) WS_2 monolayer refractive index versus wavelength, extracted from [3]. (c/d) Reflectivity map versus energy (vertical axis) and cavity length (horizontal, in units of $\lambda_C = 0.62\ \mu m$) in absence/presence of a monolayer of WS_2 (deposited on the bottom mirror, with a thickness of 0.65 nm). The grey vertical line at $L_C = 3.4\lambda_C$ in these panels represents the corresponding reflectivity spectrum represented in panel (e), where the green/red colour trace encodes the reflectivity spectrum in absence/presence of the WS_2 monolayer. (f) Extracted from panels (c,d), reflectivity minima versus energy and cavity length, revealing the polariton

mode splitting (red trace) occurring from the strong coupling between the longitudinal cavity modes and the WS_2 exciton. The green trace corresponds to panel (a), where the WS_2 monolayer is absent. The cuts of the grey vertical line with the red trace at $L_C = 3.4\lambda_C$ reveals a Rabi splitting of 20 meV, in excellent agreement with the experimental observations. (g) Reflectivity map versus energy (vertical axis) and in-plane momentum (horizontal), revealing the polariton dispersion, this simulation is obtained for $L_C = 3.4\lambda_C$, similar as that reported in the experimental dispersion relation shown in Fig. 1 of the main text. (h) Extracted from panel (g) and from the reflectivity of the empty cavity (not shown here), reflectivity minima versus energy and cavity length, revealing the dispersion relation of the upper and lower polariton modes (red trace) and the parabolic dispersion relation of the empty cavity (green trace).

Supplementary Note 3. Description of the coupled oscillator model

To describe the upper and lower polariton resonances, we employ a standard two-coupled-oscillators model:

$$\begin{bmatrix} E_{ex} & V/2 \\ V/2 & E_{cav} \end{bmatrix} \begin{bmatrix} X \\ C \end{bmatrix} = E \begin{bmatrix} X \\ C \end{bmatrix} \tag{3.1}$$

where E_{ex} and E_{cav} denote the energies of the exciton and cavity modes, respectively, and V is the normal mode splitting. For the lower polariton branch the Hopfield coefficients X and C are given by:

$$|X|^2 = \frac{1}{2}\left(1 + \frac{E_{cav} - E_{exc}}{\sqrt{(E_{cav} - E_{exc})^2 + V^2}}\right) \tag{3.2}$$

$$|C|^2 = 1 - |X|^2 \tag{3.3}$$

Their squared amplitudes $|X|^2$ and $|C|^2$ quantify the exciton and cavity photon fractions. The eigenenergies of the upper and lower polariton branches are obtained by solving the eigenvalue problem:

$$E_{UP,LP}(k_{||}) = \frac{1}{2}\left(E_{ex} + E_{cav} \pm \sqrt{V^2 + (E_{cav} - E_{exc})^2}\right) \tag{3.4}$$

Supplementary Note 4. Control on exciton-polariton detuning in the open cavity

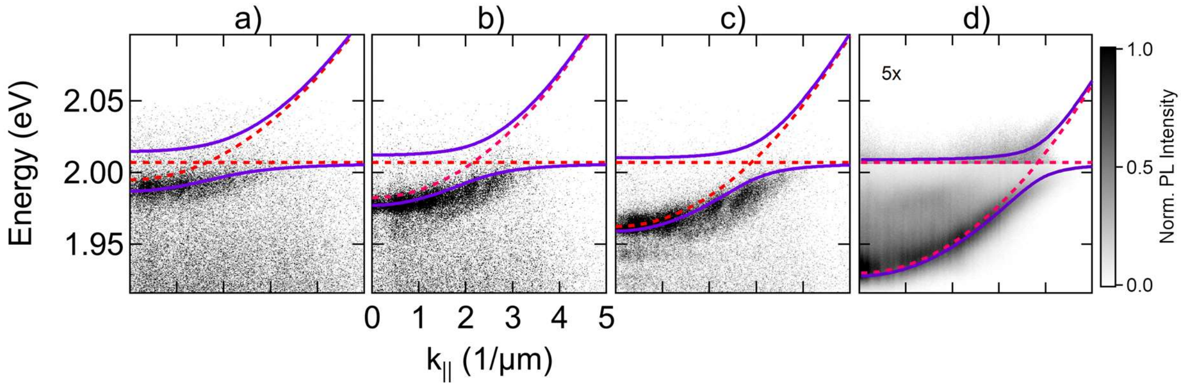

Supplementary Figure 7. Exciton-polariton detuning in the planar open cavity. Momentum-resolved photoluminescence spectra on the planar cavity for increasing mirror separation from left to right. The uncoupled excitonic (WS_2) and photonic modes (planar cavity) are represented by dashed lines. Solid violet lines indicate the upper (UPB) and lower polariton branch (LPB). d) The data above the exciton energy (E_x = 2.007 eV) is magnified by a factor of 5 to enhance the visibility of the upper polariton branch. For all spectra a Rabi splitting of 25 meV was obtained and an individual detuning of -12.0/-24.5/-42.0/-77.0 meV, respectively. The acquisition conditions for the different figures are slightly

different, for panels (a-c) the pump power is ~340 μW and an integration time of 15 s, for the last panel (d) we obtain a better signal-to-noise ratio, here we excite with a lower power ~100 μW and integrate for 600 s.

Structure	*|Δ| (meV)*	*|X|² (%)*	*|C|² (%)*	m_{LP}/m_e	***Supplementary Figure***
Planar cavity	12.0	28.4	71.6	$7.76*10^{-5}$	**7a**
Planar cavity	24.5	15.0	85.0	$5.53*10^{-5}$	**7b**
Planar cavity	42.0	7.0	93.0	$4.82*10^{-5}$	**7c**
Planar cavity	77.0	2.4	97.6	$4.48*10^{-5}$	**7d**

Supplementary Table 1. Various detunings in the planar cavity. Detuning Δ , calculated Hopfield coefficients and lower polariton mass of the planar cavity (a,b,c,d) for different mirror separations.

Structure	s_{BM1}			p_{BM1}			Fig.
	|Δ| (meV)	|X|² (%)	|C|² (%)	|Δ| (meV)	|X|² (%)	|C|² (%)	
Chain, D/A=1.7	93.1	1.7	98.3	67.3	3.1	96.9	**3a(I)**
Chain, D/A=1.7	108.8	1.3	98.7	81.5	2.2	97.8	**3a(II)**
Chain, D/A=1.7	127.9	0.9	99.1	99.1	1.5	98.5	**3a(III)**
Chain, D/A=1.7	151.1	0.7	99.3	122.2	1.0	99.0	**3a(IV)**
Chain, D/A=1.7	180.9	0.5	99.5	153.6	0.6	99.4	**3a(V)**

Supplementary Table 2. Various detunings in the photonic lattice cavity. Detuning Δ and calculated Hopfield coefficients of the 1D linear chain (D=5 μm, D/A=1.7) for the s-(s_{BM1}) and p-band(p_{BM1}) for different mirror separations.

Supplementary Note 5. Experimental results on single- and double-trap structures

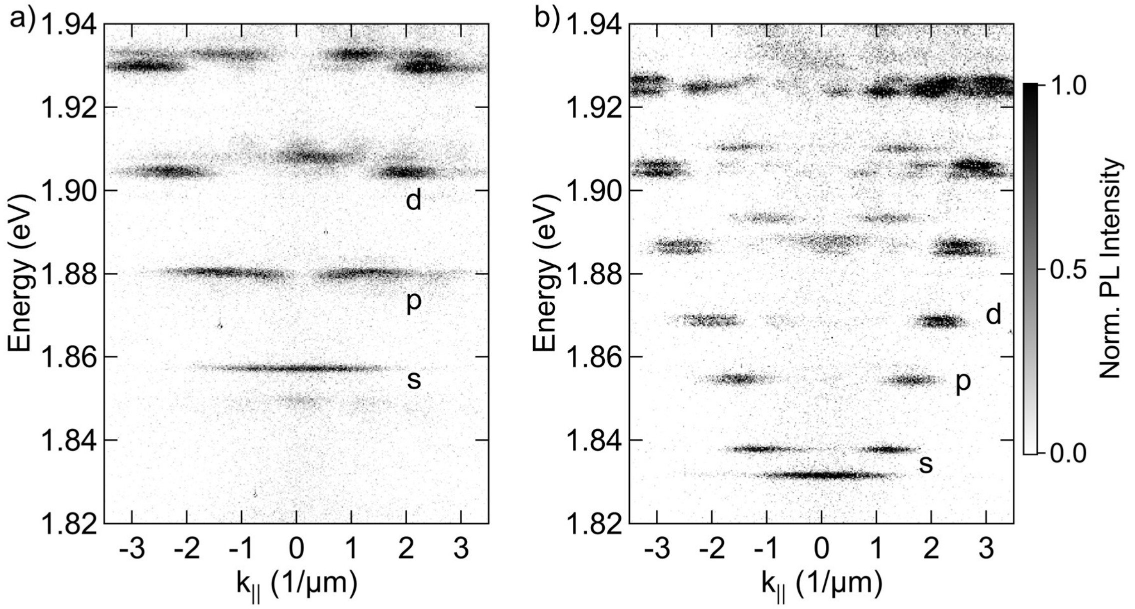

Supplementary Figure 8. (left/right) Polariton photoluminescence map versus energy and in-plane momentum, showing the dispersion relation of a single lens (left, 6 μm diameter) and a molecule (right, 5.5μm diameter and overlap D/A=2.5). The photoluminescence intensity is encoded in a false colour scale (see normalised scale on the right side). The excitation conditions are the same as those described in the main text, with a pump power of ~190 μW and 300 s integration time.

Supplementary Note 6. Bloch Modes within the mean-field model with effective potential

In order to determine the energy-momentum band structure of polaritons in the 1D lattice, we calculate first the Bloch modes in the mean-field approximation, where the geometry of the chain is represented by an effective potential in two transverse (x and y) directions. The mean-field approach is valid in the vicinity of a longitudinal resonance and it requires that the respective longitudinal mode profile between the mirrors (z-direction) is fixed. Then, in the first approximation of the perturbation theory, it is possible to reduce the three-dimensional problem to respective two-dimensional one (x- and y-) by separating the longitudinal mode profile (z-direction).

Appling this mean-field approach we solve the following eigenvalue problem for the energy $E(k_{||})$ of the Bloch mode with the Bloch vector $\boldsymbol{k} = k_{||}\vec{\mathrm{e}}_x$:

$$E(k_{||})\begin{Bmatrix} p_b\,(\mathbf{r},k_{||}) \\ e_b\,(\mathbf{r},k_{||}) \end{Bmatrix} = \hat{L}(k_{||})\begin{Bmatrix} p_b\,(\mathbf{r},k_{||}) \\ e_b\,(\mathbf{r},k_{||}) \end{Bmatrix}, \tag{6.1}$$

where the functions $p_b\,(\mathbf{r},k_{||})$ and $e_b\,(\mathbf{r},k_{||})$ describe the amplitude distributions of the photonic and excitonic components of the Bloch modes in real space defined by the resonator $\mathbf{r} = \{x,y\}$. The main matrix in Eq. (S5.1), describing the single-particle coupled states of excitons and photons, is given by the expression

$$\hat{L}(k_{||}) = \begin{pmatrix} \omega_C^0 + V(\boldsymbol{r}) - \frac{\hbar}{2m_C}\left(\vec{\nabla}_\perp + ik_{||}\,\vec{\mathrm{e}}_x\right)^2 & \Omega_R \\ \Omega_R & \omega_E^0 - \frac{\hbar}{2m_E}\left(\vec{\nabla}_\perp + ik_{||}\vec{\mathrm{e}}_x\,\right)^2 \end{pmatrix}. \tag{6.2}$$

In the model above, the quantities ω_C^0 and ω_E^0 represent the energies of bare photons and excitons ($\hbar\omega_E^0 = 2.025\,eV$), respectively. The photon-exciton coupling strength is given by the parameter Ω_R which defines the Rabi splitting $2\hbar\Omega_R = 20meV$ between coupled excitons within TMDC and photons of the cavity mode. The kinetic energy of polaritons is characterized by the effective mass $m_C \approx 5.1 \cdot 10^{-6} m_e$ (m_e free electron mass) which defines transport properties of the intracavity photons. The effective exciton mass is $m_E = 10^5 m_C$.

The periodic array with the period a is modeled by the two-dimensional potential $V(\boldsymbol{r})$ and consists of spatially-overlapping polaritonic traps. A single, separate trap has an ellipsoid-shaped potential profile defined as the real part of the function:

$$V(x,y) = V_0\left(\sqrt{1-\left(\frac{x}{R_x}\right)^2-\left(\frac{y}{R_y}\right)^2}-1\right)\Bigg/\left(\sqrt{1-\left(\frac{d}{2R_y}\right)^2}-1\right), \tag{6.3}$$

with the potential depth $V_0 = 170\,meV$ and the spatial size d. R_x and R_y describe the radii of the hemispheric dimples in the upper mirror which can be slightly different in both transverse directions.

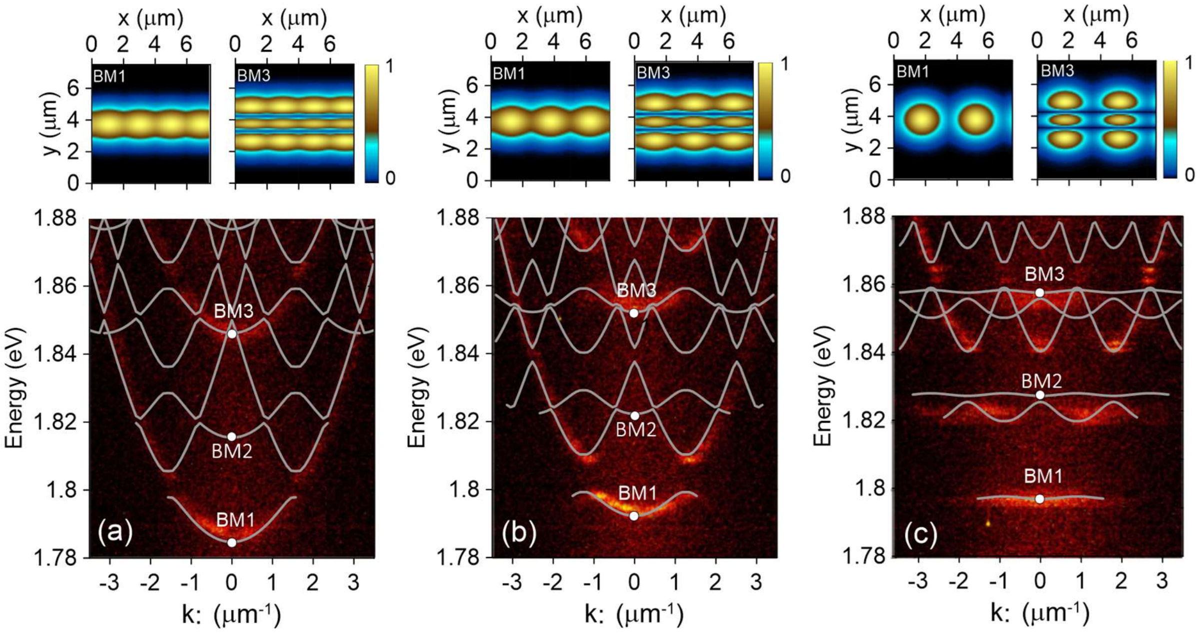

Supplementary Figure 9. (a-c) Comparison of the theoretical results (solid lines) and experimental measurements (colour map) of the energy-momentum spectrum for three different chain periods, namely, for $2\mu m$ (a), $2.5\mu m$ (b) and $3.5\mu m$ (c). The insets show profiles of the Bloch modes with one (BM1) or three field maxima (BM3) in transverse to chain (y) direction. The best theoretical fits have been obtained for the following system parameters: (a) $\hbar^2/2m_C = 7.2\, meV\cdot\mu m^2$, $\hbar(\omega_C^0 - \omega_E^0) = 261\, meV$, $d = 4.5\mu m$, $R_x = 19.8\mu m$, $R_y = 14.4\mu m$; (b) $\hbar^2/2m_C = 7.5\, meV\cdot\mu m^2$, $\hbar(\omega_C^0 - \omega_E^0) = 255.3\, meV$, $d = 4.8\mu m$, $R_x = 20.2\mu m$, $R_y = 15.4\mu m$; (c) $\hbar^2/2m_C = 8.0\, meV\cdot\mu m^2$, $\hbar(\omega_C^0 - \omega_E^0) = 255.3\, meV$, $d = 4.8\mu m$, $R_x = 18.2\mu m$, $R_y = 15.4\mu m$.

Supplementary Figure 9 shows energy-momentum spectra of the Bloch-modes calculated for three different periods of the chain (see also main text, Fig. 2). The higher order modes in transverse to the chain (y-) directions form its own sub-bands, depicted by BM1 and BM3. The respective mode profiles are shown in insets to Supplementary Figure 9. It is worth mentioning that, due to the spatial symmetry, the modes with odd number of field maxima only can be visible in the present experiment.

Supplementary Note 7. Bloch Modes beyond mean-field model

The mean-field approximation with an effective potential discussed above allows for calculation of mode profiles in the transverse plane of the cavity (x- and y- directions). However this approach becomes inaccurate if several longitudinal modes (z-direction) are involved into dynamics. For instance this model describes inadequately the cavity-length dependency of the p-band width, discussed in the main text of the manuscript.

To overcome this discrepancy we prepare further mode analysis based on a direct calculation of Maxwell equations in the given refractive index environment, including substrate, Bragg reflectors and the cavity. To avoid unnecessary computational efforts we consider a two-dimensional geometry assuming that the system is homogeneous in the transverse to the chain (y-) direction.

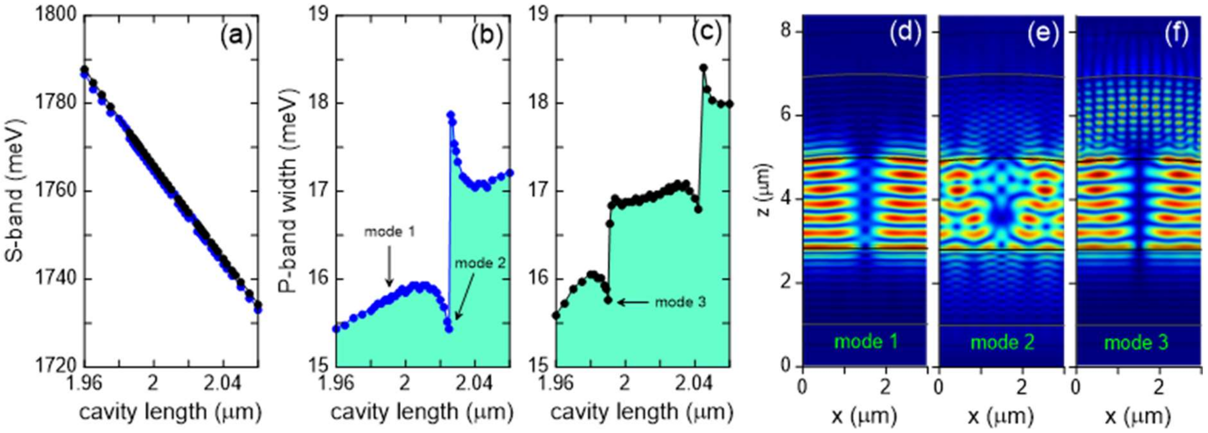

Supplementary Figure 10. Bloch modes of the open cavity calculated beyond the mean-field approximation (FEM-method). (a) The energy of the bottom of the S-band versus the separation between upper and lower Bragg mirrors (cavity length L.) for the chain period $a = 3\mu m$. Panels (b) and (c) show the widths of the p–band depending on the cavity length for TM- (b) and TE- (c) polarised modes, respectively. The width of P-band is defined as the energy difference between modes in the middle ($k_{\|} = 0$) and at the edge ($k_{\|} = \pi/a$) of the Brillouin zone. Panels (d), (e) and (f) show the Bloch mode profiles at the edge of the Brillouin zone for different cavity lengths. (d) TM-polarized mode (y-component of the electric field is zero) for $L = 1.99\mu m$. (e) TM-polarized mode for $L = 2.025\mu m$. (f) TE-polarised mode (electric field polarized in y-direction) for $L = 1.99\mu m$.

By using the finite-element method (FEM, COMSOL Multiphysics) we calculated the energy of s-band versus the cavity length, shown in Supplementary Figure 10 (a). As expected the energy of the s-band experiences a red shift of about $43\ meV$ by increasing the cavity length by $100\ nm$. Then we define the width of the p-band as difference between energies of the p-band Bloch modes in the middle and at the edge of the Brillouin zone. The calculated dependence of the P-band width on the cavity length is plotted in Supplementary Figure 10, for TM- (b) and TE- (c) polarized modes.

The electric-field profile of the typical p-band Bloch mode (TM) is plotted in Supplementary Figure 10(d). Further numerical analysis shows that the respective cavity mode can couple to the Bragg mirror modes resulting in their hybridization (see mode profiles in Supplementary Figures 10(e) and (f)). Such interaction between cavity and mirror Bloch modes explains the strong jumps in the p-band width within a small range of modification of the cavity length (see the peaks in Supplementary Figures 10(b) and (c)). We believe that this mode hybridization mechanism can explain the observed drop of the p-band width over more than 5 meV by changing the cavity length in the range of several nanometers (see Fig. 4 in the main text of the manuscript).

Supplementary References

1. Castellanos-Gomez, A. *et al.* Deterministic transfer of two-dimensional materials by all-dry viscoelastic stamping. *2D Mater.* **1**, 301–306 (2014).
2. Knopf, H. *et al.* Integration of atomically thin layers of transition metal dichalcogenides into high-Q, monolithic Bragg-cavities: an experimental platform for the enhancement of the optical interaction in 2D-materials. *Opt. Mater. Express* **9**, 598 (2019).
3. Li, Y. *et al.* Measurement of the optical dielectric function of monolayer transition-metal dichalcogenides: MoS 2 , Mo S e 2 , WS 2 , and WS e 2. *Phys. Rev. B* **90**, 205422 (2014).
